# Progress in the application of sustained-release drug microspheres in tissue engineering

**DOI:** 10.1016/j.mtbio.2022.100394

**Published:** 2022-08-13

**Authors:** Lian Ruan, Mengrong Su, Xinyun Qin, Qingting Ruan, Wen Lang, Minhui Wu, Yujie Chen, Qizhuang Lv

**Affiliations:** a College of Biology & Pharmacy, Yulin Normal University, Yulin, 537000, China; b Guangxi Key Laboratory of Agricultural Resources Chemistry and Biotechnology, Yulin, 537000, China

**Keywords:** Microspheres, Drug release, Microfluidics, Polysaccharide, Protein, Tissue engineering

## Abstract

Sustained-release drug-loaded microspheres provide a long-acting sustained release, with targeted and other effects. There are many types of sustained-release drug microspheres and various preparation methods, and they are easy to operate. For these reasons, they have attracted widespread interest and are widely used in tissue engineering and other fields. In this paper, we provide a systematic review of the application of sustained-release drug microspheres in tissue engineering. First, we introduce this new type of drug delivery system (sustained-release drug carriers), describe the types of sustained-release drug microspheres, and summarize the characteristics of different microspheres. Second, we summarize the preparation methods of sustained-release drug microspheres and summarize the materials required for preparing microspheres. Third, various applications of sustained-release drug microspheres in tissue engineering are summarized. Finally, we summarize the shortcomings and discuss future prospects in the development of sustained-release drug microspheres. The purpose of this paper was to provide a further systematic understanding of the application of sustained-release drug microspheres in tissue engineering for the personnel engaged in related fields and to provide inspiration and new ideas for studies in related fields.

## Graphical abstract

**Figure:**
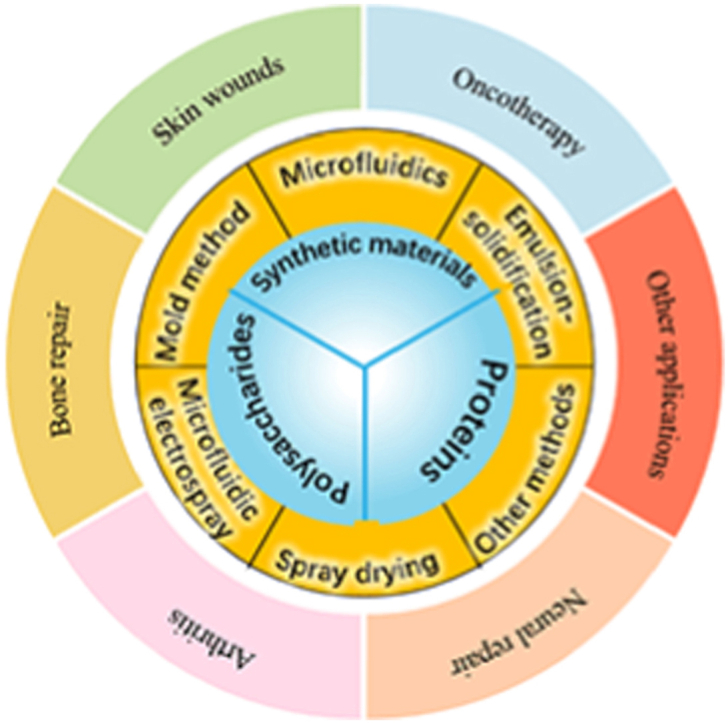

## 1 Introduction

Drug therapy is one of the most important methods used to treat diseases. However, in the process of traditional drug therapy, there are a series of problems, such as short drug action time, large fluctuations in drug concentrations, and the risk of side effects. For example, in conventional chemotherapy, commonly used in the treatment of cancer, the mechanism of action involves toxic chemotherapy molecules interacting with and damaging DNA, thereby inducing tumor cell death [1,2]. This treatment has the disadvantages of drug toxicity, a fast degradation rate, low specificity, and limited targeting [3]. At present, the development of drug delivery systems (DDSs), also known as new drug delivery systems, has attracted attention. This refers to the different methods of delivering various therapeutic drugs in the prevention and treatment of diseases, with unique advantages in terms of drug resistance, low toxicity, the possibility of double delivery, advances in chemotherapy, etc [[4], [5], [6], [7]]. New drug delivery systems have a short development cycle and relatively low cost, making them a good choice for drug development in the biomedical field. New drug delivery systems include slow- and controlled-release drug delivery systems, nanodrug delivery systems, targeted drug delivery systems, etc.

Sustained-release drug carriers are an important type of drug delivery system, with the aim of reducing or overcoming the problems caused by traditional drug therapy, and causing drugs to act slowly in the blood, which plays an important role in drug therapy. The term “sustained-release drug carrier” generally refers to a system in which the drug can slowly enter the blood, in order to reduce the concentration of the drug in the blood, thus providing drugs with a sustained long-term release. Sustained-release drug carriers include skeleton-type sustained-release preparations, film-coated sustained-release preparations, osmotic pump controlled-release preparations, and controlled-release microcapsules and microspheres [8].

A microsphere is a micron-sized sphere consisting of a continuous phase of one or more mixed and dissolved polymers, in which drugs and other components are dispersed or dissolved in a matrix [9]. Microspheres can be divided into solid microspheres, double-layer microspheres, hollow microspheres [10], and porous microspheres [11,12]. In a solid microsphere, the entire microsphere is dense. Although solid microspheres can indeed achieve long-acting drug release, they also have problems such as high initial concentration explosions, long drug release times, and low efficiency of encapsulation; therefore, solid microspheres are not currently widely used [13,14]. In order to solve the problems of solid microspheres, solid microspheres can be modified through the use of polymers to coat the microsphere products to reduce the initial burst concentration. Such microsphere products are called double-layer microspheres, also known as double-walled microspheres. The use of polymers as microsphere product shells can not only improve their drug carrying capacity, but can also reduce the initial burst of the drug [15]. Hollow microspheres, with a hollow inner section and a uniform shell, can be loaded drugs and other small molecular substances can be used. It has been proven that optimized hollow adhesive microspheres have good floating and sustained-release characteristics due to the buoyancy of their inner hollows [16]. For example, hollow microspheres synthesized by Li et al. using the self-loading method showed an improved encapsulation rate, diameter size, and conductivity, and improved the utilization of drugs in organisms [17]. In porous microspheres, the surface of the shell has many voids. Porous microspheres are prepared by adding a pore-forming agent to solid microspheres. These characteristics of these microspheres include a large specific surface area, low density, controllable porosity, etc [18]. There are three main types of pore-forming agents used for the preparation of porous microspheres: permeable, gas-forming, and extractable agents. Wang et al. used porous microspheres, solid microspheres, and hollow microspheres to conduct drug release tests in vitro, and found that the encapsulation rate and final cumulative drug release of porous microspheres were significantly better than those of the other two types of microspheres, indicating that porous microspheres have great potential in sustained-release systems [19]. In addition, the preparation of magnetic microspheres through the introduction of magnetic substances has demonstrated good drug-loading and sustained-release ability [20], as well as super paramagnetism, which enables drugs to be transported to the target via a magnetic field [21]. Microspheres are mainly administered orally, intravenously, or through subcutaneous implantation, intraperitoneal injection, etc. Compared with traditional drugs, microspheres can significantly improve patient compliance [22]. Long-acting sustained-release drugs with a low frequency of administration and few drug side effects have been widely used in tissue engineering, biomedicine and other fields, and this area has become a research hotspot in recent years [23,24].

Because of the uniform shape and size of the microspheres and their other unique features, microspheres can adsorb or diffuse ions, extracellular molecules, and drugs in the regeneration process, and can be used to store drugs and as bioactive molecular carriers [25], and they therefore have good development prospects and have attracted wide attention. Therefore, we have conducted a systematic review of the application of sustained-release drug microspheres in tissue engineering in recent years. First, we summarize several processing methods of microspheres, including the emulsification method, the microfluidic method, the template method, etc. Second, we summarize the synthetic materials used for microspheres, such as PLGA, PCL, PLA, etc. Third, the application of microspheres in various diseases is summarized. Finally, based on the known developments and our personal understandings, we summarize the shortcomings of microspheres in tissue engineering and discuss future prospects in the development of microspheres. The purpose of this paper is to provide the personnel engaged in related fields with a further systematic understanding of the application of sustained-release drug microspheres in tissue engineering, and to provide inspiration for further contributions to the development of related fields.

## 2 Processing methods of microspheres

In tissue engineering applications, there are many processing methods used for microspheres. Different processing methods are selected according to the properties of the materials, the slow-release drug properties of the microspheres, and their purpose. Microspheres can be processed via the emulsification method, the microfluidic method [26], the template method, the microfluidic electrojet method [27], and other common processing methods (Table 1).

**Table 1:** Summary table of preparation technology and application of microspheres.

Preparative ​technique	Merits	Demerits	Application	Microsphere-drug ​combination	Ref.
Emulsion-solidication	Large-scale production, simple operation	Wide particle size	Oncotherapy Neuralrepair	1. TPP/chitosan/PLGA-NGF Microspheres 2. 5-Fluorouracil loaded biodegradable magnetic Microspheres 3. Polysaccharidebased porous microsphere (PPM)	[88,188,189]
Microfluidics	Unique analytical performance, smaller volume, good monodispersity	Low production efficiency	Oncotherapy	5-Fu loaded CS microspheres	[30,35]
Mold method	Low cost, simple operation	Complex synthesis process	Protein separation	Monodisperse porous silica microspheres	[58,62,63]
Microfluidic electrospray	Uniform size and good monodispersity	Low production efficiency	Skin wounds	Novel drug-loaded methacryloyl chondroitin sulfate (CSMA) microspheres	[67, 196]
Spray drying	Simple and stable	Limited range of polymers	Gastropathy	Chitosan-based microspheres	[75]
Self-assembly method	Simple production technology, mild reaction conditions, and suitability for hydrophilic and hydrophobic drugs	Size and monodispersity need to be improved	Oncotherapy	Paclitaxel-loaded silk fibroin nanospheres	[78]
Phase separation method	For water-soluble drugs, easy to prepare in batches	Difficult to remove organic solvents	Arthritis	Loaded sPL sustained-release microspheres	[206]
Membrane emulsification method	Particle size uniformity, mild conditions, simple equipment and energy saving	Small dispersion flux, low production efficiency	The organic-inorganic hybrid composites	Biopolymer-inorganic hybrid microspheres	[87]

### 2.1 Emulsion-solidication

**Fig. 1:**
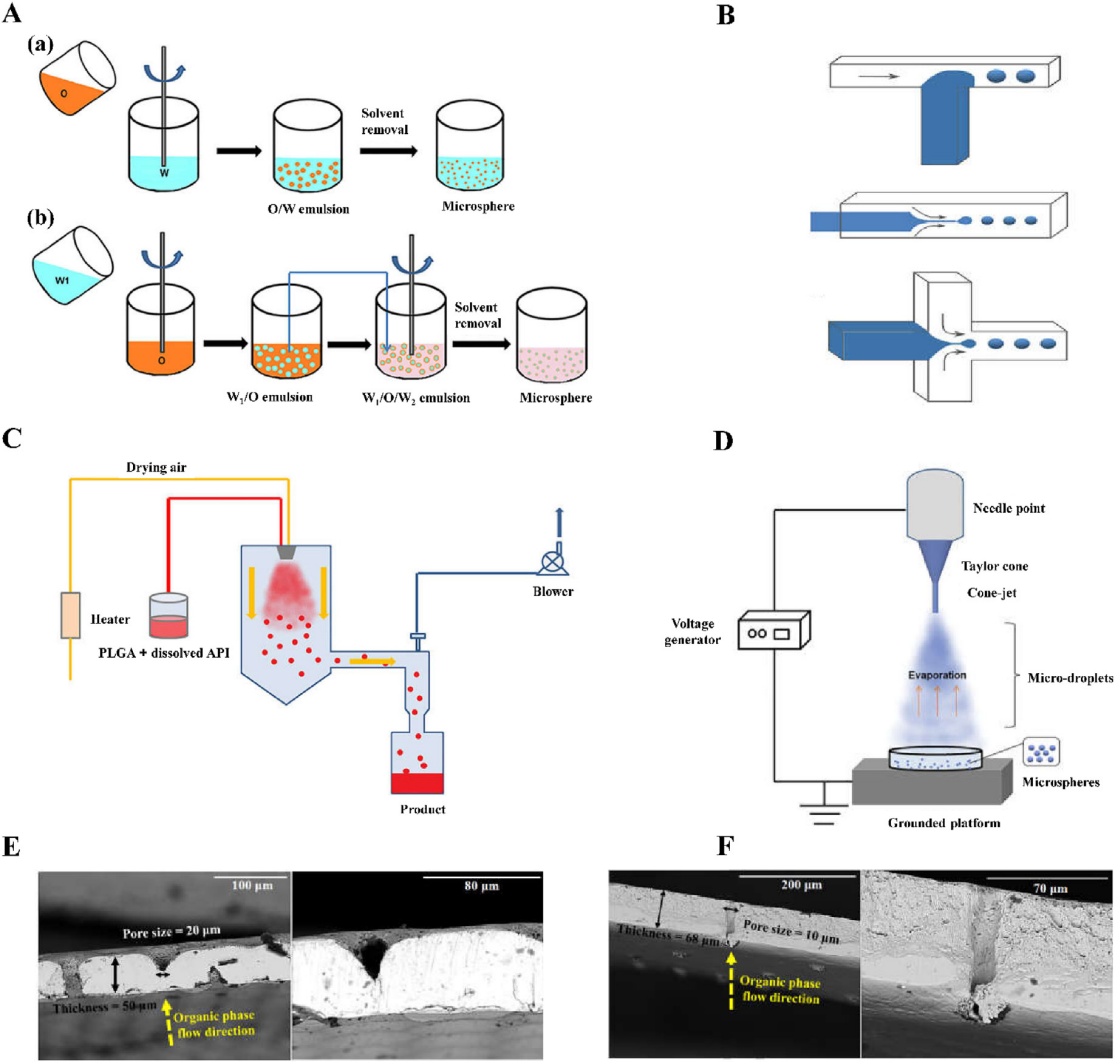
Schematic of fabrication process and scanning electron microscope of membrane section. A Schematic of PLGA microspheres prepared via (a) single emulsion and (b) double emulsion. Reproduced with permission from Ref. [29]. Copyright 2020, Taylor and Francis. B Schematic of microfluidic devices for making PLGA microspheres: T-Junction microfluidic device, co-flow microfluidic device, flow-focusing microfluidic device. Reproduced with permission from Ref. [29]. Copyright 2020, Taylor and Francis. C Schematic diagram of the spray drying process. Reproduced with permission from Ref. [29]. Copyright 2020, Taylor & Francis. D Schematic of the electrospray process for preparing PLGA microspheres. Reproduced with permission from Ref. [29]. Copyright 2020, Taylor and Francis. E Scanning electron microscope (SEM) images of membrane cross sections at various magnifications: whole nickel (Ni) membrane. Reproduced with permission from Ref. [85]. Copyright 2016, Elsevier. F Scanning electron microscope (SEM) images of membrane cross sections at various magnifications: stainless steel (SS) ringed membrane. Reproduced with permission from Ref. [85]. Copyright 2016, Elsevier.

Emulsification [28] is one of the commonly used methods to prepare microspheres. The emulsifying method is affected by many factors, such as the emulsifying equipment, emulsifying temperature, emulsifying time, and stirring speed. Emulsions can be divided into the O/W type, W/O type, O/W/O type, W/O/W type, etc. During the preparation of microspheres, different emulsions can be selected depending on the nature of the wrapped drug (Fig. 1A) [29,30].

The emulsifying crosslinking method can be divided into the emulsifying physical crosslinking method and the emulsifying chemical crosslinking method. The difference between the two is that the physical crosslinking method is based on the unique physical properties of the material itself, whereas the chemical crosslinking method is based on the connection of chemical bonds. For example, in the emulsification physical crosslinking method, an aqueous alkaline urea solution is used to dissolve chitosan, and after emulsification, the in situ thermogel behavior unique to the physical properties of the alkaline chitosan solution is used to prepare non-toxic chitosan microspheres that can be dissolved by lysozymes [31]. In the emulsified chemical crosslinking method, konjac or chitosan microspheres are prepared through chemical bonding between the negative group of sodium tripolate and the amino group in chitosan [32]. Zhao et al. injected isoperidone microspheres prepared via emulsified solvent evaporation into mice, and these proved to be more effective in the treatment of schizophrenia, compared with mice treated with oral isoperidone [33]. The emulsified curing method refers to the preparation of an emulsion by removing the solvent to solidify the dispersed phase. This method of preparing microspheres can enable the packaging of active substances in particles that are suitable in drugs that are insoluble or indissoluble in the continuous phase, and according to the nature of the embedding material, one can choose the appropriate emulsification method to improve the coating rate of microspheres and drug loadings. However, this method involves the use of organic solvents, which do not easily maintain drug activity, and the reemulsification process is complex, which is not conducive to industrial production. The emulsification dispersion method involves the addition of surfactants in two incompatible liquids to form emulsions and the use of chemical crosslinkers to solidify the preparation of microspheres. This is generally combined with the chemical crosslinking method. The use of this method to prepare microspheres does not require special equipment, is suitable for laboratory production, and is suitable for water-soluble and fat-soluble drugs.

### **2.2** Microfluidics

Microfluidics, also known as microfluidic chip technology or lab on a chip, is a technology that enables precise control and manipulation of microscale fluids, especially submicron structures [[34], [35], [36], [37]]. Microfluidic processing of microspheres mainly depends on the microchannel system used. In the microchannel system of a microfluidic, there are drip, backflow, laminar flow, jet flow, extrusion flow and wave flow phenomena. These fluid phenomena can be regarded as the symbols and the important characteristics of microfluidics. In relation to microfluidics, there are two methods used to form droplets: the active method and the passive method [38]. In the active method an external force is used to drive and control the droplet generation process [39], whereas in the passive method the droplet generation process is controlled by controlling the two-phase flow rate ratio and the microtubule structure. The active method is divided into the optical drive method and the electric drive method. The optical drive method refers to the method of using a Gaussian beam enhanced by means of an isoelectron body to generate two-phase droplets [40]. Common electric drive methods include the electrowetting method and the dielectric electrophoresis method. The electrowetting method uses the applied electric field to change the interface free energy between the fluid and the contact surface, so that the surface is infiltrated by the fluid. After the electric field is turned off, the surface becomes hydrophobic, and the fluid that has infiltrated on the surface breaks from the liquid storage tank and forms droplets [41]. In the dielectric electrophoresis method, fluid is pulled out of the liquid storage pool to form droplets, and the frequency and size of the applied electric field can determine the sizes of the droplets [42,43]. There are four main methods for the preparation of droplets via passive methods: the flow focused, co-flow, T-junction and step junction methods (Fig. 1B) [44].

#### 2.2.1 Flow focused

The term “flow focused” refers to the fact that in a microfluidic system, the mobile phase flows parallel to the dispersed phase in the pipeline, and finally the mobile phase is squeezed by the shear force of the dispersed phase to form droplets [45]. The flow focused method is the simplest and most basic of the four methods, and can be improved according to different needs. Some studies have taken bubble generation as the object and determined that a periodic Taylor bubble generation has three modes of shear, transition, and wetting, and the conical structure of co-axial flow can enhance the stretching and squeezing effect on droplets, reducing droplet size and increasing the frequency of droplet generation [46].

#### 2.2.2 Co-flow

Co-flow refers to a microfluidic system in which a pipeline gathers three flow paths, and the mobile phase and the dispersed phase converge in the cross pipeline. The dispersed phase is squeezed and fractured by the mobile phase, symmetrically distributed up and down, and thus generating droplets [47]. For example, Mitropoulos et al. used a co-flowing capillary device to prepare microspheres and submicron spheres containing silk fibroin with polyvinyl alcohol as the continuous phase and silk fibroin solution as the dispersed phase. The diameter of the microspheres could be controlled by controlling the concentration of polyvinyl alcohol and silk fibroin solution and the liquid flow rate ratio [48].

#### 2.2.3 T-junction

The T-junction method is a common method to form liquid droplets in microfluidics. It refers to the fact that the insoluble fluid of two phases meets at the vertical intersection of the T-junction. Under the joint action of shear force and pressure, the dispersed phase is truncated by the mobile phase and droplets are generated [49]. Experiments have proven that the size of the droplets mainly depends on the structural shape of the T-junction, especially its height and width, whereas the mobile phase and dispersed phase control the density and frequency of droplets [50].

#### 2.2.4 Step junction

Step junction method is generally composed of widened channels and steps [30]. The step junction method enables multiple nozzles to be arranged in parallel on a large scale to achieve high-throughput production of droplets. Moreover, the step junction method is mainly driven by the interface tension rather than shear force. Therefore, the size of droplets has nothing to do with the flow rates of continuous and dispersed phases, and mainly depends on the geometry of channels in the device. Therefore, high droplet productivity, a small diameter variation coefficient and good monodispersity can be maintained ect [51,52]. However, droplets tend to gather near the nozzle, which will affect the formation of new droplets, reduce the formation rate of droplets, and lead to the expansion of droplet size distribution [53,54]. A large amount of research has been conducted to address this problem. For example, Schular et al. proposed a centrifugal step emulsification method that uses the density difference and centrifugal force to cause droplets to leave the nozzle directly and not to accumulate near the nozzle, thus achieving the high-frequency production of monodisperse droplets [55].

Microfluidics have the advantages of unique analytical performance, smaller volume, fewer samples, and low energy consumption, and have great development prospects in biomedicine and other fields. For example, Han et al. prepared polyvinyl alcohol microspheres by combining droplet microfluidic technology with a physical crosslinking method. The droplet size was able to be controlled by adjusting the injection flow rate of the two-phase fluid and the width of channel. This method produced microspheres with high efficiency and good monodispersity [56].

### 2.3 Mold method

The mold method of preparing composite microspheres is not only widely used in the literature on composite materials, but it is also one of the important methods to process microspheres. The mold method is one of the common methods for preparing hollow microspheres. The mold method involves taking a specific substance as a template, and then wrapping the material on the surface of the template via physical and chemical methods. Finally, the template is removed by means of solvent dissolution, high-temperature calcination, cleaning, and other methods to obtain hollow microspheres. The mold method can be divided into the soft template [57] and hard template methods, according to the composition and characteristics of the template used. The difference between the two is that the soft template forms a dynamically balanced cavity, and the material can enter and exit through the cavity wall in the form of diffusion, whereas the hard template forms a static pore, and the material can only enter into the pore from the opening. The soft template method uses liquid droplets, bacteria, bubbles, etc., rather than solid particles. Compared with hard template method, the soft template method has the advantages of easy template removal, simple operation, short operation time, low cost, and environmental friendliness [58]. Ju et al. prepared rambutan-like zinc oxide layered hollow microspheres using hydroxymethyl starch as a soft template [59]. The hard template method, also known as nano-casting [60,61], takes inorganic materials as templates, and the materials used for the template are mainly silicon dioxide, metal oxide, or a metal oxide skeleton. Microspheres prepared by means of the hard template method have a regular structure and size, but it is difficult to remove the template, and the method involves a complex synthesis process, and the method has a relatively high cost, is time-consuming, and would be difficult to use widely in industry, etc. [62] For example, porous silica microspheres, prepared by crosslinking glycidyl methacrylate and ethylene glycol dimethacrylate, were used as hard templates to prepare porous silica microspheres with ethyl orthosilicate by means of the sol-gel method [63].

### 2.4 Other methods

#### 2.4.1 Microfluidic electrospray

Microfluidic electrospray technology is considered a promising and multifunctional technology, capable of using small amounts of liquid and providing an efficient and simple method for the preparation of microspheres [64,65]. Microfluidic electrospray devices are simply composed of capillaries (Fig. 1C) [66]. Although microfluidic electrospray technology is not widely used at present, it has also been applied to the production of microspheres. For example, Lei et al. prepared novel angiogenic microspheres to treat thin endometrium using microfluidic electrospray technology based on hyaluronic acid methacrylate hydrogel with a porous structure, and proved that the microspheres had uniform size, good monodispersity, and drug release ability [67]. Microfluidic electrospray technology was used to prepare magnetic biohybrid microspheres composed of an agarose–hyaluronic acid mixture, which could be used to isolate and deliver growth factors secreted by *Escherichia coli* to treat chronic trauma [68]. Yang et al. used microfluidic electrospray technology to prepare biomass-carrying stem cell microcapsules for the treatment of bone defects and applied them in animal experiments [69]. Studies showed that the microcapsules prepared using this method had good compatibility and therapeutic effects, and had broad development prospects in the tissue engineering and medical fields.

#### 2.4.2 Spray drying

When preparing microspheres using the spray drying method [70], the drug and shell material solution are mixed; the polymer solution is sprayed in inert hot air to evaporate; and the polymer solution is diffused, dried, and precipitated, so that the polymer shrinks into shells and the drug is wrapped to form solid particles (Fig. 1D) [[71], [72], [73], [74]]. A peristaltic pump is an important part of a spray dryer. This method has the advantages of creating no pollution, providing high precision in liquid regulation, good sealing performance, and low shear force. Moreover, the speed of the peristaltic pump can affect the encapsulation rate of the microspheres, which is conducive to constant liquid transportation in the coating process. For example, Zhang et al. used chitosan as the carrier and extracts of Panax notoginseng, Codonopsis pilosula, and Atractylodes macrocephala as the raw materials to prepare drug microspheres that could protect the gastric mucosa via the spray drying method. The method of preparation of such microspheres was simple and stable [75]. Mouez et al. prepared sticky chitosan microspheres by wrapping verapamil hydrochloride via the spray drying method, showing that the spray drying method had the advantages of a small burst release, a long duration, and improved bioavailability [76].

#### 2.4.3 Self-assembly method

The self-assembly method refers to the process in which nano-scale particles can spontaneously form regular structures [77]. The self-assembly method mainly relies on hydrogen bonds, intermolecular covalent bonds, the electrostatic force, and other sub-valent bond forces to assemble polymers. The self-assembly method displays simple production technology, mild reaction conditions, and suitability for hydrophilic and hydrophobic drugs, but the size and dispersion of the microspheres need to be improved. For example, Chen et al. prepared silk fibroin nanospheres containing the anticancer drug paclitaxel via the self-assembly method, dissolving paclitaxel in ethanol, mixed with silk fibroin solution, and freezing the mixture at 20 ​°C to prepare the microspheres [78]. Cheng et al. used the self-assembly method to prepare silk fibroin microspheres, and then mixed the microspheres with a chloroform solution to prepare microcapsules with controllable size and adjustable permeability [79].

#### 2.4.4 Phase separation method

The phase separation method refers to a process in which the drug and carrier material and mixed, and then the two phases are separated under specific conditions, and the carrier material is separated out to wrap the drug and form microspheres. The phase separation method can be divided into the water phase separation method and the oil phase separation method, according to the solubility of the wrapped material in water, and the water phase separation method can in turn be divided into the single condensation method and the complex condensation method [[80], [81], [82]]. Single condensation is phase separation that occurs due to non-electrolytes, whereas complex condensation is phase separation that occurs due to the neutralization of two solutions with opposite charges. The phase separation method is mainly used for water-soluble drugs, displaying good pelletization performance for hydrophilic drugs, and these drugs are easy to prepare in batches. For example, Chen et al. prepared a novel triptolide acetate microsphere using PLGA as the carrier via the phase separation method, which was characterized by a high encapsulation rate and a low initial release rate [83]. However, many organic solvents are used in the preparation process, meaning these microspheres are difficult to remove, aggregate easily, and are difficult to produce on a large scale.

#### 2.4.5 Membrane emulsification method

The membrane emulsification method refers to the process of pressurizing the dispersed phase liquid and forming a monodisperse emulsion through the microporous membrane with the same small pore size. The droplet size reaches an appropriate range and, under the action of force, it disengages from the membrane hole and enters the continuous phase. The core of membrane emulsification method is the membrane, the surface of which is filled with pores. The main types of membranes include Shirasu porous glass (SPG) membranes, polymer membranes, stainless steel membranes, etc. (Fig. 1E and F) [84,85] and SPG membranes are the most common type [86]. Compared with the traditional emulsification method, the membrane emulsification method has the characteristics of uniform particle size, milder conditions, the use of simple equipment, and energy savings, but its small dispersion flux leads to low yield, which limits its application. For example, via the combination of membrane emulsification and biomimetic mineralization, researchers prepared gelatin/alginate/silica hybrid microspheres, and microspheres for a model enzyme, immobilized to glycerol dehydrogenase, and thereby improved the protective effects of its resistance to pH, and its storage and recycling stability improved, compared with free glycerol dehydrogenase [87].

## 3 Microsphere processing materials

The materials used for the preparation of microspheres are mainly divided into two categories, namely, synthetic polymer materials (such as poly (lactic-*co*-glycolic acid [88], polycaprolactones [89], etc.) and natural polymer materials (such as polysaccharide polymers [90], and protein polymers). Polysaccharides mainly include alginate [91], hyaluronic acid [92], chitosan [93] and agarose [94], etc. Proteins include fibrin [95], bovine serum albumin [96], gelatin [97] and so on. The microspheres prepared from different materials have different biological and chemical characteristics. Natural materials have the advantages of good biodegradability and the degradation products are safe and non-toxic [98], but they are not easy to control in terms of the size, preparation process and stability of microspheres. Compared with natural materials, synthetic materials have the advantages of good biocompatibility, good mechanical properties, stable properties and controllable drug release [99]. However, some polyester materials need to add crosslinking agents or adhesives during the preparation of microspheres, resulting in low drug embedding rate and low in vivo adhesion.

### 3.1 Synthetic materials

Many polymerizable small molecules can be used to form macromolecules with specific functions, such as polylactic-*co*-glycolic acid, polylactic acid, polyethylene glycol diacrylate, N-isopropyl acrylamide, etc. The prepared drug sustained release microspheres with the use of synthetic polymer materials, not only in vivo has good biodegradability and biocompatibility, and its degradation products are non-toxic and can be absorbed and utilized by organisms and other advantages, so the synthetic polymer materials in the drug sustained release microspheres are widely used, and the most mature for synthetic materials research is polylactic acid-hydroxylactic acid copolymer and polylactic acid [100].

#### 3.1.1 PLGA

**Fig. 2:**
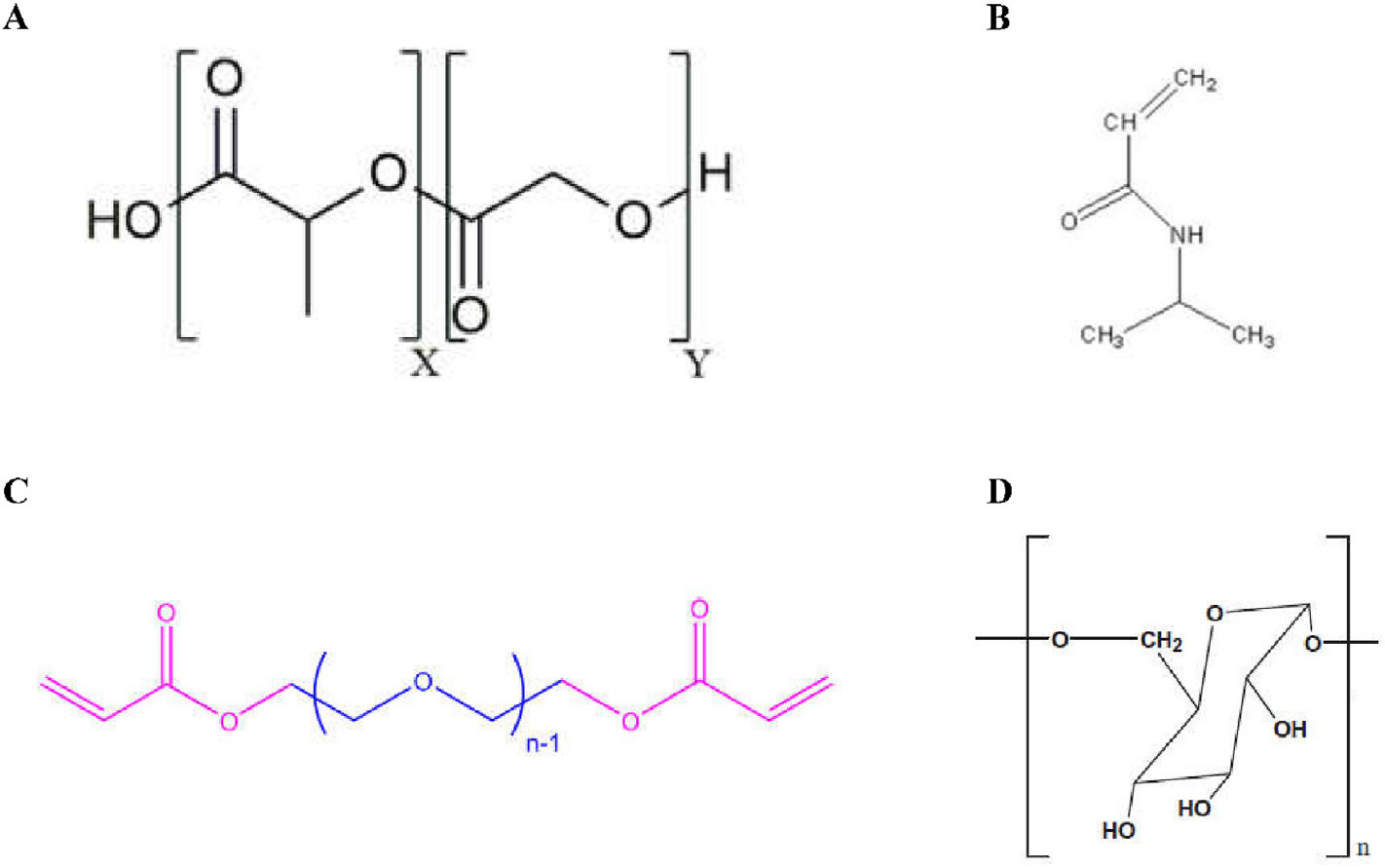
Structural diagrams of PLGA, PEGDA, NIPAM and polysaccharides. AChemical structure of the poly (D,L – lactide-co-glycolide) (PLGA) co-polymer. Reproduced with permission from Ref. [183]. Copyright 2019, Frontiers. B Structural diagram of NIPAM. Reproduced with permission from Ref. [125]. Copyright 2015, Springer. C Repeat unit of PEGDA (n ​= ​43) used in this study. Blue and pink regions represent the PEG segment and the acrylate cross-linker,respectively. Reproduced with permission from Ref. [118]. Copyright 2019, American Chemical Society. D Structural formula diagram of polysaccharides. Reproduced with permission from Ref. [128]. Copyright 2018, Elsevier. (For interpretation of the references to colour in this figure legend, the reader is referred to the Web version of this article.)

PLGA is a biodegradable functional polymer organic compound made from the polymerization of two monomers, lactic acid and hydroxyacetic acid (Fig. 2A) [101]. PLGA has been certified by the FDA and officially included in the United States Pharmacopoeia as a pharmaceutical excipient. PLGA has been widely used in biomedical and modern industrial fields due to its advantages of good biocompatibility, long-acting sustained release and targeting, a long drug action time, safety and non-toxicity, and good encapsulation and film formation [102,103]. PLGA is used as carrier material to prepare nanocapsules, microspheres, gels, etc., among which microspheres are becoming increasingly widely used. Microspheres can provide a long-term sustained release of drugs at the site of action, protecting drugs and improving the utilization of the drug biology, so they have great advantages in clinical practice. PLGA nanocarriers have been widely used because of their safety for the human body [[104], [105], [106]]. For example, PLGA nanocarriers, modified with polyethylene glycol (PEG), can circulate in the body for a longer time, significantly improving passive targeted therapy for tumors [107]. In addition, Zhou et al. prepared PLGA microspheres loaded with mesoporous silica nanoparticles (MSNs) using a capillary three-phase microfluidic device [108]. The results showed that the drug release behavior of PLGA microspheres could be effectively regulated by adding mesoporous silica nanoparticles into PLGA microspheres.

#### 3.1.2 PCL

Polycaprolactone is a kind of classical synthetic degradable polymer material [109]. Polycaprolactone is insoluble in water but soluble in a variety of organic solvents. It can be completely degraded into carbon dioxide and water in the natural environment, and the degraded products can be absorbed or discharged by the body. Because of its non-toxicity, good biocompatibility, and biodegradability, it has been widely used in the controlled release of drugs [110]. Based on this, Nyoke et al. prepared biodegradable polycaprolactone microspheres for drug delivery and combination engineering applications using freeze-drying technology and double emulsification at different PCL concentration ratios [111]. In addition, Dhanka et al. prepared polycaprolactone microspheres (PCL MPs) and MTX-PCL microspheres (MTX-PCL MPs) using the oil-in-water emulsion solvent volatilization method [112]. The results showed that in vitro, The MTX release of MTX-PCL MPs in phosphate buffer with pH ​= ​7.4 showed controlled release characteristics, which also indicates that the microspheres can be used for drug delivery.

#### 3.1.3 PLA

Polylactic acid (PLA) is prepared via the artificial polymerization of lactic acid as a raw material. The original material of PLA is starch, which is easily decomposed into carbon dioxide and water by microorganisms in nature after use. It not only does not produce pollution in the production process, but also has good biodegradability and biocompatibility [113,114]. At the same time, PLA can be used as a drug agent and can be broken down into carbon dioxide and water in the human body, thus displaying no toxicity or side effects for the human body. This material can also be biodegradable. PLA polymer materials can be used as a carrier for a specific drug made. After mixing, when microspheres have arrived at the corresponding parts of the body, the polylactic acid slowly decomposes into carbon dioxide and water. Meanwhile, the drug is gradually released at the appropriate site. Compared with the direct administration of drugs without PLA as a carrier, the microspheres can protect the drugs, increase the local drug concentration, and increase the therapeutic index. For example, Li et al. constructed VEGF-PLA nano-sustained-release microspheres, which showed good biocompatibility, degradability, and other physical and chemical properties after their mixed injection with SVF cells and human fat particles [115]. The results showed that the survival and quality of transplanted adipose tissue could be improved by adding VEGF-PLA nano-sustained-release microspheres and SVF cells. Frounchi et al. used FTIR, XRD, VSM, SEM, and drug release testing to study magnetite and polylactic acid/polyethylene glycol drug-loaded microspheres [116]. The results showed that the control of the drug release depended on the combination of diffusion and PLA, and also followed the non-Fick mechanism. As a plasticizer of PLA, polyethylene glycol can not only promote drug release, but can also release more of the drug in the presence of a magnetic field.

#### 3.1.4 PEGDA

Polyethylene glycol diacrylate, a hydrophilic polymer [117], is widely used in biomedicine and other fields (Fig. 2C) [118], as part of a process in which an acrylate crosslinking agent is used to synthesize polyethylene glycol diacrylate on the existing polyethylene glycol hydrogel [119]. For example, Peng et al. prepared 5-fluorouracil biocompatible PEGDA microspheres with a monodisperse particle size distribution by means of in-situ photopolymerization of a microfluidic device for the study of the sustained release of drugs [120]. The results showed that PEGDA microspheres were able to be used as a long-term and stable carrier of a 5-fluorouracil chemotherapy-controlled drug delivery and screening system, which indicates that the microspheres have broad application prospects. Lin et al. used microfluidic technology to prepare chitosan/PEGDA hydrogel microspheres (CP-MSSs) with controllable size via the oil-in-water method after photo-crosslinking and physical crosslinking [121]. The results showed that cp-MSSs loaded with chondrocytes could be used for injection, and the cell viability was still high after injection. The results also indicated the potential application of these injectable chondrocell-loaded microspheres in cartilage tissue engineering.

#### 3.1.5 NIPAM

N-isopropylacrylamide can be polymerized to form a poly (N-isopropylacrylamide) organic compound (Fig. 2B). Poly (N-isopropylacrylamide) is a typical and widely used temperature-sensitive material. Its low critical phase transition temperature is 25 ​°C-32 ​°C, which is close to human body temperature [122]. Meanwhile, the PNIPAM polymer contains both hydrophilic amide group and hydrophobic isopropyl group components. However, due to the poor softness and high brittleness of N-isopropylacrylamide monomer polymer at room temperature, it is necessary to modify and graft NIPAM onto the microsphere to give the microsphere the function of controlling the drug release [123,124]. For example, Zhang et al. grafted the temperature-sensitive monomer NIPAM to cellulose microspheres in a low-temperature microreactor by means of in situ free radical polymerization, and studied the swelling behavior and drug release ability of CFN-PNIPAM composite microspheres [125]. The results showed that the microspheres containing PNIPAM showed a controllable drug release rate. Furthermore, porous CFN-PNIPAM composite microspheres can be used as a novel material to control the drug release.

### 3.2 Polysaccharides

Polysaccharides are a class of natural polymers with complex molecular structures and a wide variety of molecular structures, with molecular weights ranging from thousands to millions, which are widely distributed in nature and play an important role (Fig. 2D) [126,127]. They are dehydrated and condensed by multiple monosaccharide molecules. Polysaccharides are indispensable biological macromolecules in life activities and are found widely in animals, plants and microorganisms [[128], [129], [130]]. For example, cellulose and peptidoglycan are among the important components of the cell wall, starch and glycogen store animal and plant energy, etc. Studies have shown that polysaccharides have a variety of biological activities and have been used in medical research and development, such as anti-tumor, anti-oxidation, anti-virus, anti-coagulation, immunity enhancement, and hypoglycemia research, etc [131]. Polysaccharides show rich biological activity, high biocompatibility, excellent degradability, safety, and no side effects, and have good application prospects and broadly available sources, making them important participants in the natural extraction of drugs [132]. With the development of science and technology, new agents have been gradually used in the research on preparations involving polysaccharides, such as liposomes, nanoparticles, microcapsules and microspheres, polymer micelles, vesicles, etc. Among these, the research on sustained-release drug microsphere carriers has been developing for more than 40 years to date. Compared with traditional drug delivery systems, microspheres can reduce the adverse reactions of drugs, improve the stability of drugs, enable the slow release of drugs, and improve the targeting of drugs, so as to increase their efficacy. Common polysaccharides for the preparation of sustained-release microspheres include the use of alginate, hyaluronic acid, chitosan, agarose, cellulose and glucan, etc.

#### 3.2.1 Alginate

Alginate is a natural polysaccharide compound. It is a linear polymer composed of (1,4) -β -l-mannuronic acid (M) and its stereostructure (1,4) α-D-Gurolonic acid(G), bonded by β-1,4 and −1,4-glycosidic bonds. It includes the homopolymer M block (MM), homopolymer G block (GG), and heteropolymerized alternate blocks (MG). Due to its good biocompatibility, biodegradability, hydrophilicity, adhesion ability, and other excellent characteristics, alginate has shown potential application value in the fields of tissue engineering and biomedical materials [133]. Studies have shown that alginate microspheres have the advantages of oral safety, non-toxicity, and good biocompatibility, so their usage can effectively improve the utilization and effectiveness of drugs. For example, Alpdemir et al. encapsulated synthesized superparamagnetic iron oxide nanoparticles (SPIONs) together with the anti-tumor drug sorafenib in alginate microspheres, and according to their drug release studies, sorafenib was released from the microspheres over 8 ​h, indicating that the microspheres were able to prolong the time of drug release, improve the efficiency of the drug, and also meaning that microspheres are expected to be used in tumor treatment [134]. In addition, Sanchez-Ballester et al. used the Ca:Mg ionic gel method with different proportions to prepare heteroionic calcium-magnesium alginate microspheres, and studied the dynamic swelling of the microspheres, and found that when the pH was 1.2 and 7.2, the amount of Mg ions was added, and the swelling rate was also increased, and the study also showed that increasing the amount of Mg ions in the beads could increase the rate of drug release [135].

#### 3.2.2 Hyaluronic acid

Hyaluronic acid was first isolated from the vitreous humor of a bull's eye by Meyer and Palmer in 1943 [136]. In addition, hyaluronic acid is found in almost all biological fluids and tissues, and at the same time it is also one of the main physiological components of the extracellular matrix (ECM) in all connective tissues of the body [137]. Hyaluronic acid is an acidic macromolecule mucopolysaccharide that is widely used in clinical applications because of its unique advantages of viscoelasticity, adaptability, biocompatibility, and non-immunogenicity [[138], [139], [140]]. For example, in recent years, hyaluronic acid, as a carrier of drugs, has been used to form compounds with other drugs, providing a slow-release and playing a targeting role, so that the combined drugs can be released in a targeted manner, thus improving the efficacy of drugs [141]. Among these compounds, microspheres are also a kind of drug carrier, playing the same role. For example, Yan et al. prepared gelatin/hyaluronic acid (Gel/HA) composite microspheres via the emulsion-coagulation method, using glutaraldehyde (GA) as a crosslinker to study and analyze free gelatin/HA and gelatin/HA icariin microspheres [142]. The results showed that the release of icariin in gelatin/HA microspheres decreased with the increase in the content of the crosslinking agent, GA, and the crosslinking time, and when the content of icariin in the microspheres increased, its cumulative release also increased, indicating that the gel/HA can be used as a sustained-release microsphere carrier and the microspheres can adjust the release rate of icariin. Athamneh et al. prepared lung drug carriers using alginate and alginic acid/hyaluronic acid as raw materials, using emulsifying gel technology and a supercritical CO_2_ drying method [92].

#### 3.2.3 Chitosan

**Fig. 3:**
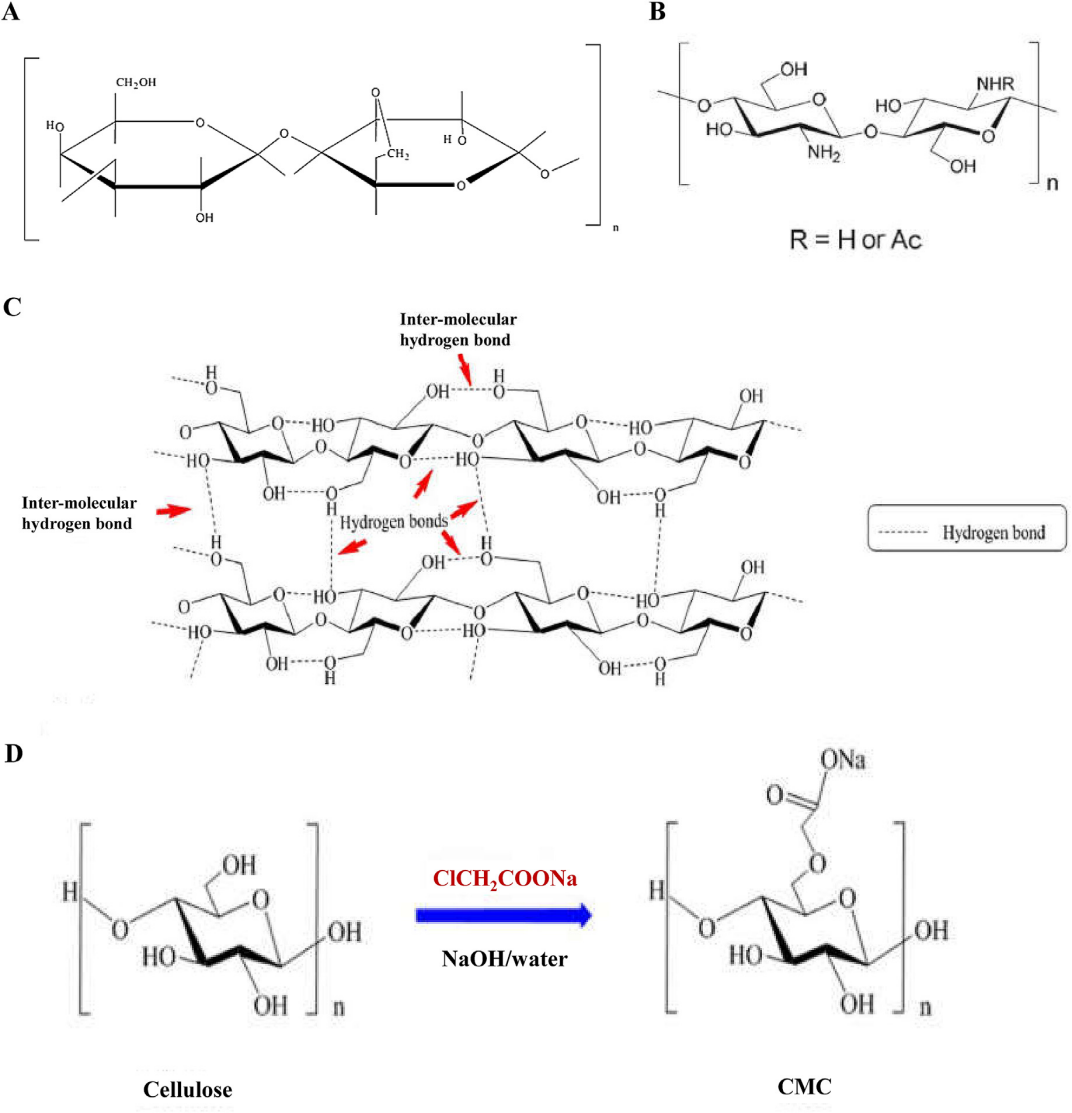
The structure of chitosan, agarose and cellulose and the schematic diagram of CMC synthesis. A Structure of agarose. Reproduced with permission from Ref. [146]. Copyright 2014, Elsevier. B Chitosan. Reproduced with permission from Ref. [93]. Copyright 2021, Elsevier. C Schematic representation of intra and inter-molecular hydrogen bonding pattern of cellulose structure. Reproduced with permission from Ref. [150]. Copyright 2021, Elsevier. D the preparation and structure of CMC. Reproduced with permission from Ref. [150]. Copyright 2021, Elsevier.

Chitosan is the product of partially removing acetyl groups from chitin. It contains a large number of active amino and hydroxyl groups (Fig. 3B), so it has good biocompatibility, degradability and antibacterial properties, and other functions. Chitosan can only be dissolved in acidic solutions, but it is difficult to dissolve in water, alkaline solutions, and organic solvents, so chitosan solutions are usually acidic. In addition, chitosan drug-loaded microspheres have become the focus of research because of their targeting ability, sustained release, and biodegradability. For example, Liu et al. prepared mercapto chitosan (CS-TBA)/CaCO_3_ double microspheres by means of the ionic gel method. The double microspheres combined the advantages of CS-TBA, which improved the water solubility and the mesoporous structure of CaCO_3_ [143]. The results showed that the CS-TBA/CaCO_3_ double microspheres could be used as a dual-dosing system, thus having potential application prospects in tissue engineering and regenerative medicine. Ramos-Torres et al. prepared hybrid microspheres of palygorskite (PAL) nano-clay and crosslinked chitosan (QS) via an emulsion method [144].

#### 3.2.4 Agarose

Agarose is a natural polymer polysaccharide material, of which the basic disaccharide units include 1,3-linked β-D-galactose and 1,4-linked α-L-3,6-dehydrated galactose (Fig. 3A). Agarose is currently widely used in biomedicine and bioengineering. In addition, it has unique properties such as hydrophilicity, long-term stability, non-toxicity, and porosity, making agarose an ideal synthetic material for the preparation of microspheres [145]. It has demonstrated good biocompatibility, porosity, and hydrophilicity with microspheres [146,147]. Zhang et al. prepared chitosan-agarose microspheres loaded with berbamine using water-in-oil emulsification technology [148]. Under the optimal conditions, the packing rate of the microspheres was 84.57%, the drug loading was 8.44%, and the swelling rate at pH 7.4 was higher than that at pH 1.2 These findings suggest that berbamine was released in a pronounced manner from the microspheres. In addition, Hasan et al. conducted in vitro and in vivo osteogenesis performance tests to assess the biocompatibility of BCP-CSD-agarose complex microspheres [94]. In addition, their in vivo experimental results also showed that the composite microspheres exhibited good bone regeneration effects.

#### 3.2.5 Cellulose

Cellulose is a rich and widely distributed natural polymer, which occurs mainly in the form of glycans in the plant cell wall (Fig. 3C) [149]. Cellulose is a renewable biological resource, and displays several advantages, such as low price, good biocompatibility, rapid degradation, relatively low environmental pollution, recyclability, etc. Because cellulose microspheres have good biocompatibility, good biodegradability, safety and non-toxicity, etc., they are widely used in medicine and biology. In addition, when cellulose microspheres are used as drug carriers, they have the function of targeting and providing a sustained and controlled release. Wei et al. prepared aerogel microspheres and possible pathways of microspheres using chitosan and cellulose as raw materials, and described a nuclear/shell structure of aerogel microspheres and microspheres for the controlled release of drugs (Fig. 3D) [150]. In addition, Yan et al. used novel porous CaCO_3_ microspheres with hollow nanoparticles (NPs) with diameters of 15–35 ​nm as templates [151]. The results showed that sustainable hollow cellulose nanoparticles (NPs) were able to regulate the sustained-release effect of CaCO_3_ microspheres.

#### 3.2.6 Glucan

Glucans are naturally biodegradable polysaccharides linked by glycoside bonds, which can be divided into α-dextran and β-dextran according to the type of glucan bond. Because of its biodegradability, biocompatibility, and gel properties, dextran can be used as a substitute for plasma in clinical practice, so it is a good raw material for the preparation of microspheres. Dextran microspheres are polymer microspheres prepared via a crosslinking reaction between dextran and a crosslinking agent. In addition, glucan microspheres have a very broad application prospects in many fields, such as the biomedical field, tissue engineering, etc. Among these, the use of glucan for drug controlled release carriers is widely occurring at present, and the underlying principle of this method is to wrap the glucan and make a microsphere around the drug, so that the drug plays a role in targeting and providing a sustained release. Hou et al. synthesized glucan microspheres using reversed phase suspension polymerization, and then prepared octyl dextran microspheres via a reaction and freeze drying method with ethylhexyl glycidyl ether, using doxorubicin (DOX) as a drug model to observe the ability of poporooctyl dextran microspheres to carry drugs [152]. The results showed that the drug loading rate increased with an increase in the microsphere/drug ratio, whereas the packaging rate decreased; in short, the octyl dextran microspheres had the potential to act as an effective delivery system to control the drug release.

#### 3.2.7 Chondroitin sulfate

**Fig. 4:**
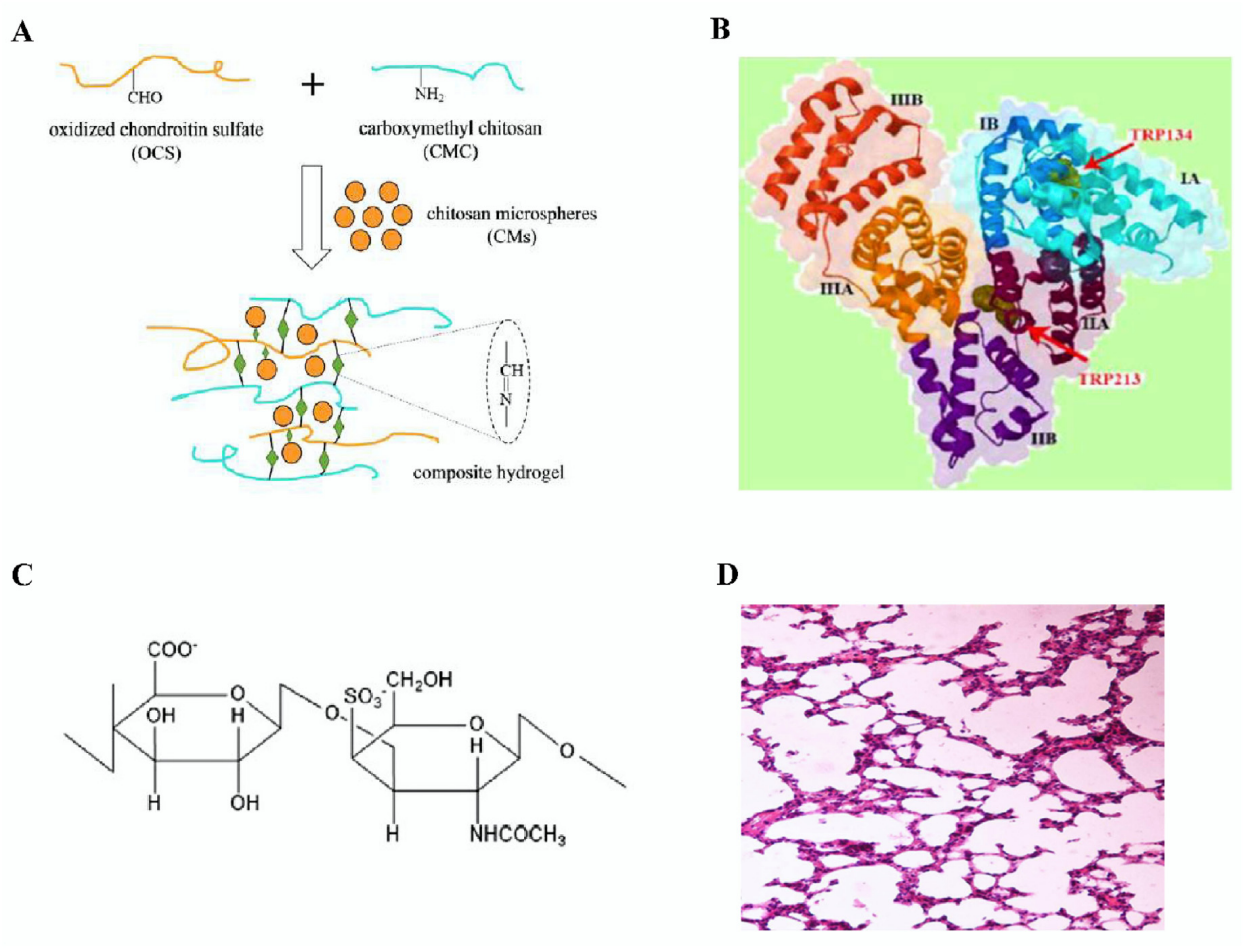
Structure diagram of BSA, CS, gel scaffold and albino mouse lung. A Reaction scheme to show a preparation of CMs embedded CMC-OCS composite gel scaffold (CMs/gel). Reproduced with permission from Ref. [154]. Copyright 2017, Elsevier. B Ribbon representation of BSA structure. Reproduced with permission from Ref. [160]. Copyright 2016, Elsevier. C CS. Reproduced with permission from Ref. [154]. Copyright 2017, Elsevier. D Cytoarchitecture of albino mice lung (azithromycin solution). Reproduced with permission from Ref. [163]. Copyright 2016, Springer.

Chondroitin sulfate is a class of glycosaminoglycans that are covalently linked to the formation of proteoglycans on proteins, which are widely present in the extracellular matrix and the cell surface of most mammalian tissues, and are a component of cartilage. Its content is related to the species of animals and the age of animals, and it has a variety of biological functions, such as regulating growth factors, promoting cartilage growth, and accelerating wound healing. In recent years, chondroitin sulfate microspheres, as a new kind of biomaterial, have attracted wide attention because of their good bioactivity, biodegradability, and biocompatibility. For example, Cellet et al. used the ultrasonic microemulsion polymerization method to encapsulate hydroxyapatite nanocrystalline whiskers in chondroitin sulfate (CS) microspheres (Fig. 4C), and the results showed that these complex microspheres could play a role in sustained release of drugs [153]. Ming et al. prepared bovine serum albumin (BSA)-loaded chitosan-based microspheres (CMs) via the emulsion crosslinking method, and then embedded them into CMC-OCS hydrogel to prepare CM/hydrogel composite scaffolds (Fig. 4A), which demonstrated that the complex can be used as an injectable drug and cell delivery system in cartilage tissues [154].

### 3.3 Proteins

Proteins are polypeptides formed by multiple amino acids in a “dehydrated and condensed” manner, and the polypeptides are then folded to form polymer compounds with a certain spatial structure. Proteins play an extremely important role in the growth and development of living organisms, which is not only the main component of living organisms, but also the basis of life activities. It has been widely used in the biomedical field because of its advantages of a wide range of sources, non-toxic and even beneficial to the human body. At present, great progress has been made in the research of long-acting preparations of protein drugs, especially the encapsulation of protein polymers and drugs into microspheres, so that drugs can be slowly released in the body and effectively improve the utilization rate of drugs. Common proteins used to prepare drug sustained-release microspheres are fibrin, bovine serum albumin, gelatin, and collagen.

#### 3.3.1 Fibrin

Fibrin is a water-insoluble protein transformed from fibrinogen and plays an important role in blood coagulation, in which fibrinogen is a coagulation factor. Fibrin is derived from the extracellular matrix of the blood and displays excellent plasticity and degradation [155]. Fibrin can be used as a carrier for drug transport as a synthetic material for microspheres, and, compared with traditional delivery methods, natural biocompatible fibrin microspheres offer the continuous delivery of packaged drugs and ease of degradation in the blood circulation. For example, Khan et al. studied the systematic enhancement of the antifungal activity of fibrin microspheres (AMB-fibrin microspheres) loaded with amphotericin B on cryptococcosis in Swiss albino mice. The study showed that AMB-fibrin microspheres exhibited improved chemotherapy effects compared with free AMB [156]. They found that AMB can be used to reduce the fungal burden, and has a better survival effect, and that the use of the novel amphotericin B drug fibrin microspheres enabled them not only to protect the wrapped amphotericin B, but also to effectively reduce drug-related toxicity. In addition, Yang et al. synthesized SHH-loaded chitosan microspheres (SHH/CS) embedded in fibrin scaffolds and then applied this treatment for the protection and regeneration of completely transverse spinal cord injuries in rats [157]. The results showed that SHH was able to maintain its biological activity and continue to act on the site of injury, and SHH/CS microsphere-embedded fibrin scaffolds showed good effects in nerve regeneration, tissue cavity prevention, and motor function improvement, compared with the separately wrapped fibrin scaffolds.

#### 3.3.2 BSA

Bovine serum albumin (BSA) is a globulin found in bovine serum (Fig. 4B). In addition, serum albumin is a globular protein with its most abundant content in human blood [158,159]. The combination of drugs and serum albumin affect the drugs’ metabolism, distribution, and transportation [160]. BSA and human serum albumin (HSA) have a high degree of homology and 76% structural similarity [161]. The de-solvent method is a method used to prepare microspheres. In addition, bovine serum albumin is also the most commonly used drug carrier in this method, and has been proven to be biocompatible, biodegradable, safe and non-toxic. For example, Xue et al. adopted a new kind of inverse emulsion ionic crosslinking method, involving a pH response to bovine serum albumin-loaded chitosan microspheres [162]. They tested this method under different pH buffer conditions, in terms of the drug release, and the results showed that the BSA in neutral solution-chitosan microspheres released the drug slowly, and released it rapidly in acidic solution. Ramaiah et al. prepared azithromycin albumin microspheres via emulsion polymerization, and their results showed that bovine serum albumin microspheres could be used to deliver targeted drugs, which reached high concentrations in the lungs of albino mice (Fig. 4D) [163].

#### 3.3.3 Gelatin

Gelatin is a type of protein, obtained via the partial hydrolysis of collagen. It is a macromolecular hydrophilic colloid. Because of its non-toxicity and its lack of antigenicity, it is a natural polymer carrier material that is widely used and has slow-release effects [164]. Gelatin is commonly used in food preparation [165], and it has excellent hydrophilic, degradability, high drug loading ability, and good histocompatibility characteristics, as well as other features [166]. Using gelatin as the carrier can not only improve the stability of drugs, but also reduce the toxic side effects of drugs and improve their efficacy [167]. Therefore, gelatin is often used in biomedical fields, such as in the production of drug capsules. In particular, gelatin is used in microparticles and microspheres to provide the controlled release of drugs [168,169]. In the 1960s, Tanaka et al. first developed an oral gelatin microsphere containing sulfonamide or riboflavin [170]. Clercq et al. used paclitaxel as a drug-loaded genipin cross-linked gelatin microsphere (PTX-GP-MS) for the treatment of microperitoneal carcinogenesis [171]. The results showed that GP-MS immersion in PTX nanosuspension reached maximum loading and prolonged the PTX release from PTX-GP-MS. When the dose of PTX-GP-MS reach to 27%, the PTX release within 14 days in mice was prolonged, and ultimately resulting in drug-related toxicity, and whilst there was no drug-induced in the range of 7.5–15 ​mg PTX/kg, indicating that low doses of PTX-GP-MS could serve as a dosing system to prevent the recurrence of ovarian cancer.

#### 3.3.4 Collagen

Collagen is a natural polymer material with a special triple-helix structure and a large number of carbon-containing functional groups (such as hydroxyl, carboxyl, and amide groups, etc.) on its molecular chain, and is also the most abundant class of proteins in mammals [172,173]. Collagen has many advantages, such as extensive sources, biodegradability, biocompatibility, safety, non-toxicity, etc., and is widely used in food, medicine, tissue engineering, cosmetics, and other fields [174], and has become one of the hot spots in scientific research. The current research on the field of application of collagen microspheres is very extensive. For example, Ji et al. combined PLGA microspheres loaded with BMP-2 or P24 with chitosan and nano-hydroxyapatite/collagen, and the results showed that the microspheres could release osteogenic factors well to promote osteogenesis [175]. Yang et al. prepared a collagen (COL) microsphere-based steroidal saponin (SS) formula via the solvent evaporation method using an oil-in-water double emulsion, and the results of in vitro release studies showed that the release efficiency of SS displayed an obvious slow-release effect, and the higher the content of COL, the longer the drug release time [176].

#### 3.3.5 Silk fibroin

Silk fibroin is a natural biomolecular material extracted from silk, which contains 18 kinds of amino acid residues embedded in a hydrophobic and hydrophilic interlaced structure, forming a special arrangement. Therefore, it has excellent characteristics, such as hydrophilicity, slow biodegradation, good histocompatibility, strong plasticity, etc [177]. Studies have shown that silk fibroin is an excellent drug carrier material, which can be used in the synthesis of fibers [178], hydrogels [179], microspheres, and other substances [180], mainly because it has many micro-gap structures inside it, which can be used to adsorb and fill drugs. Yu et al. freeze-thawed the hydrophilic anticancer drug floxuridine (FUDR) on the surface of silken proteins with a particle size between 210 and 510 ​nm, and the results showed that the maximum drug load was about 6.8% and the release time exceeded 2 days [181]. Sungkhaphan et al. aimed to prepare microspheres from Thai silkin (SF) blended with hyaluronic acid (HA), and the results showed that microspheres with a higher SF content had higher stability and slower degradation rates. In addition, in order to demonstrate the potential of use of adsorbed curcumin in the microspheres, it was finally shown that all SFHA microspheres were released drugs controlled manner [182].

## 4 Application of microspheres in diseases

Diseases have always been a major problem that plagues people, patients not only have to endure the pain caused by the disease, but also bear huge economic pressure, and the treatment effect of some diseases is not optimistic, so it is crucial to find a new type of treatment. Microspheres are part of a sustained-release drug carrier and are widely used in the biomedical field. In the treatment of modern diseases, drug-carrying microspheres are usually combined with compound microspheres with drugs, so as to achieve the drug sent to the lesion and slowly released there, which can effectively prolong the time of drug action and is a very effective means of treating diseases. At present, the application of microspheres in diseases mainly includes oncotherapy [183], skin wounds [68], bone repair [184] arthritis [185], and so on.

### 4.1 Oncotherapy

**Fig. 5:**
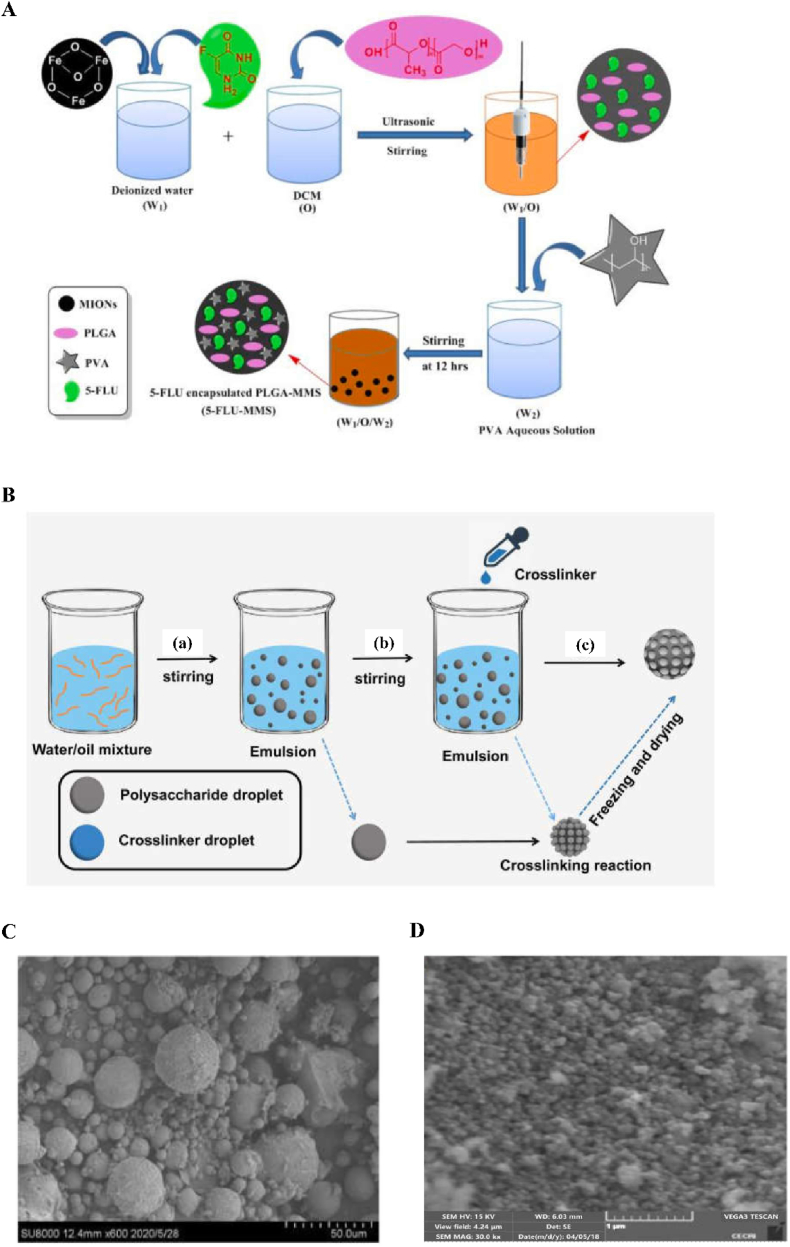
Scanning electron microscopy of 5-Flu-MMS, MS-LADed PPM and the synthesis process of 5-Flu-MMS and PPM. A Schematic illustration of preparation of 5-FLU-MMS. Reproduced with permission from Ref. [188]. Copyright 2022, Elsevier. B PPM synthesis process diagram. (a) Water and oil mixture were stirred to form inverse emulsion. Polysaccharide large emulsion droplets were evenly dispersed in the oil phase. (b) The crosslinker CaCl2 solution was added to the emulsion and formed tiny emulsion droplets. (a) Crosslinking reaction was performed between polysaccharide droplets and tiny crosslinker droplets. (c) After freezing and drying, PPMs were finally obtained. Reproduced with permission from Ref. [189]. Copyright 2021, Dovepress. C Scanning electron microscope analysis morphology of MMC-loaded PPM. Reproduced with permission from Ref. [189]. Copyright 2021, Dovepress. D SEM and EDX image of 5-FLU-MMS 4. Reproduced with permission from Ref. [188]. Copyright 2022, Elsevier.

The incidence of malignant tumors in both men and women is increasing year by year, and the mortality rate is also gradually increasing, and tumors have become one of the major public health problems seriously threatening human health [186,187]. The treatment of cancer can be carried out through four approaches: chemical drug therapy, radiation therapy, surgical treatment, and biological treatment. However, estimates of the effectiveness of treatment are not optimistic, the cure rate is low, and patients have to endure the adverse reactions brought about by the treatment for a long time, resulting in patients being troubled by huge mental pressure. Because microspheres are sustained-release drug carriers, they enable the use of targeted and long-acting sustained-release drugs, which can significantly reduce the drug burden of patients, and are one of the important means of tumor treatment. The development of slow-release drug microspheres can achieve the targeted delivery of drugs at tumor sites and reduce the generation of adverse reactions. Therefore, sustained-release drug microspheres are the key to improving the therapeutic effect of tumor treatments. For example, Ayyanaar et al. constructed drug-loaded magnetic microspheres (MMSs) loaded with the anticancer drug 5-fluorouracil (5-FlU), and the results showed that the MMSs (Fig. 5A and D) carrying the anticancer drugs could sustainably release the drugs and release the drugs at the target, thus reducing the cytotoxic effects on non-cancer tissues [188]. In addition, Wu et al. prepared polysaccharide porous microspheres (PPMs) (Fig. 5B) via reverse emulsion polymerization, and performed in vivo experiments, confirming that mitomycin C (MMC)-loaded PPMs had an inhibitory effect on malignant tumors [189]. Moreover, MMC-loaded PPMs (Fig. 5C) showed good biocompatibility, drug release ability, safety and non-toxicity, and good surface morphology, suggesting that PMMs may be potential drug carriers in tumor therapy.

### 4.2 Skin wounds

**Fig. 6:**
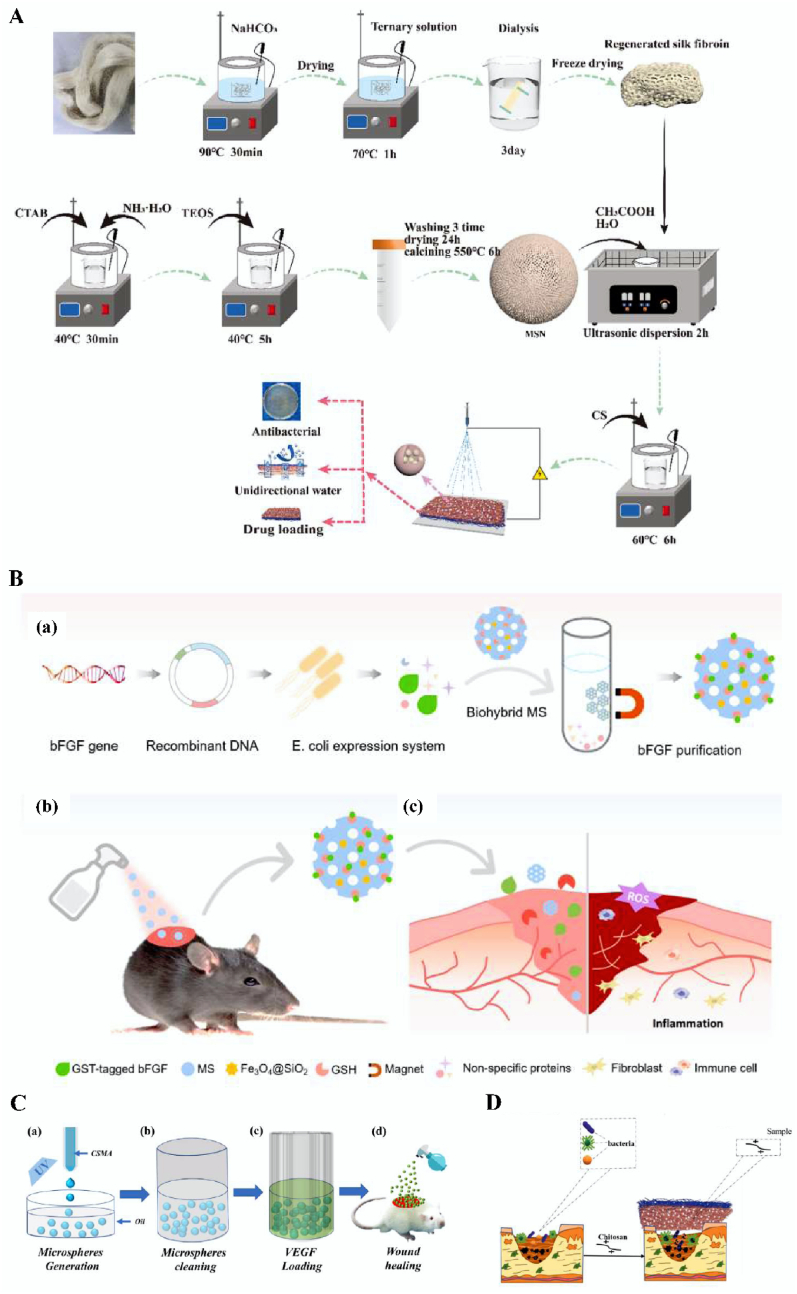
Preparation and schematic diagram of CSMA, MSN-SF/CS, MSN-SF/CS microsphere and multifunctional biohybrid microsphere system. A Diagram of the preparation process of MSN-SF/CS drug-loaded microspheres. Reproduced with permission from Ref. [197]. Copyright 2022, Elsevier. B Schematic illustration of the fabrication of multifunctional biohybrid microsphere system and its application in chronic wound healing. (a) Microspheres were designed to isolate bFGF protein which was produced using *E. coli* expression system. (b–c) Conjugated with bFGF, the biohybrid microspheres were applied for enhanced wound healing in diabetic mice. Reproduced with permission from Ref. [68]. Copyright 2021, Elsevier. C Schematic diagram of the fabrication of CSMA microspheres and their application in wound healing. Reproduced with permission from Ref. [196]. Copyright 2022, Springer. D Antibacterial schematic diagram of MSN-SF/CS microspheres. Reproduced with permission from Ref. [197]. Copyright 2022, Elsevier.

The skin is the organ with the largest epidermal area and the first barrier of the human body, and it has a very important role, protecting the human body from physical injury, chemical substances, and pathogenic microorganisms [[190], [191], [192], [193]]. In China, tens of millions of burn victims require treatment every year, as burns cause skin injuries. Skin wound healing is generally divided into three stages, namely, inflammation, proliferation, and remodeling. At any of these stages of injury, age and other factors lead to obstacles and changes in skin repair [194,195]. In recent years, instances of hard-to-heal wounds also increases, and if not handled properly, this will affect wound healing, resulting in scars, which affect the patient's appearance. Large areas of damage are also easily affected by the outside world and infections, with serious infection causing the death of the patient. Therefore, the task of skin wound repair involves enormous challenges. Microspheres have high liquid absorption rates and good biocompatibility, and have good hemostatic effects and can promote wound healing. Based on this, Zhang et al. used microfluidic electrospray to produce drug-loaded methacryloyl chondroitin sulfate (CSMA) microspheres (Fig. 6B and C) [196]. The results showed that the prepared microspheres demonstrated good biocompatibility and could significantly promote wound healing. Li et al. prepared silica nanoparticles using silk fibroin/chitosan (MSN-SF/CS) microspheres (Fig. 6A) and coated them on a silk fibroin (SF)/polycaprolactone (PCL)-polyvinyl alcohol (PVA) single-guided water composite nanofiber membrane [197]. The results showed that the microspheres demonstrated good antibacterial effects and biocompatibility (Fig. 6D), and this study stimulated and promoted the application of additional wound-healing biomaterials in clinical medicine.

### 4.3 Bone repair

**Fig. 7:**
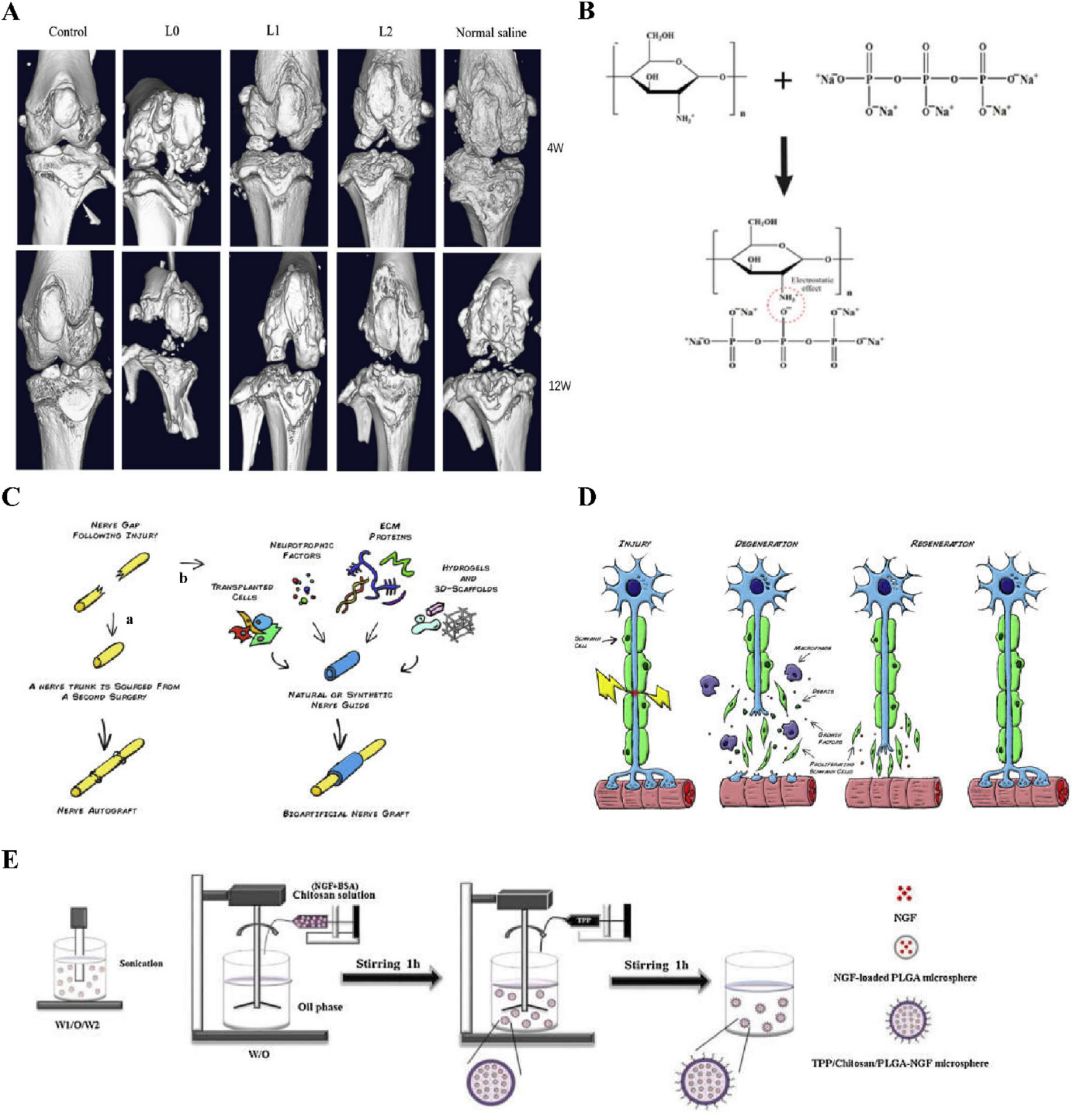
Effects of microspheres on bone, bioartificial nerve transplantation, molecular structure of crosslinked CS, Waller denaturation and preparation of microspheres by reemulsification and ion crosslinking. A Micro-CT scans showing the effect of microspheres on bone and articular cartilage injury. (Control group: Joint cavity was surgically accessed without any procedure done; L0: L0 microspheres injected after the ACLT and DMM procedures; L1, L1 microspheres injected after the ACLT and DMM procedures; L2, L2 microspheres injected after the ACLT and DMM procedures; Normal saline group: normal saline microspheres injected after the ACLT and DMM procedures). Reproduced with permission from Ref. [206]. Copyright 2021, Springer Nature. B Molecular structure of chitosan, TPP and TPP ionically cross-linked CS via electrostatic effect. Reproduced with permission from Ref. [88]. Copyright 2021, Elsevier. C Bioartificial nerve graft for nerve repair. As an alternative to autograft (a), the “gold standard” for nerve repair, the engineering of an artificial nerve graft (b) has been sought. Such graft will consist in a natural or synthetic nerve guide, which could be enriched with several factors to enhance axonal regrowth such as: 1) Transplantable cells, 2) neurotrophic factors or other pharmacological aids, 3) extra-cellular matrix (ECM) proteins and 4) hydrogels or 3D scaffolds as conduit fillers or cell/drug vehicles. Reproduced with permission from Ref. [208]. Copyright 2014, Elsevier. D Wallerian degeneration. Following injury, Schwann cells detach from the axons, start proliferating and help the recruited macrophages to clear the cellular and myelin debris. At the same time, expression of stimulating factors by SCs create a favourable environment for nerve regrowth towards the target organ. Reproduced with permission from Ref. [208]. Copyright 2014, Elsevier. E Schematic illustration of preparation of TPP/Chitosan/PLGA-NGF microspheres using a re-emulsification-ionic cross-linking method. Reproduced with permission from Ref. [88]. Copyright 2021, Elsevier. (For interpretation of the references to colour in this figure legend, the reader is referred to the Web version of this article.)

Bone defects are generally caused by trauma, infection, tumors, etc., and their regeneration is a major clinical challenge. Autologous bone transplantation is considered the “gold standard” and the most effective method of bone regeneration for the treatment of bone defects [198]. However, due to the influence of bone supply and donor site lesions, biomaterials need to be improved to match the performance of autologous bone transplantation. In recent years, the specific modification of microspheres has become a research hot spot. Microspheres with adjustable particle sizes can be used to fill in irregular bone defects, because their special micro/nano structures and porous structures are conducive to cell adhesion proliferation, thus potentially accelerating bone reconstruction (Fig. 7A). In addition, when used as drug carriers, microspheres can provide a targeted, slow release of the drug, thus prolonging its half-life [199]. Xia et al. used the ectopic osteogenesis model to study a chitosan microsphere delivery system, and their structural analysis showed that the drug delivery system could significantly enhance the induction and promotion of the ectopic osteogenesis of recombinant bone morphogenetic protein, providing basic data for the clinical application of a chitosan/recombinant bone morphogenetic protein microsphere delivery system [200]. In addition, Calasans-Maia et al. used alginate-coated minocycline nanocrystalline carbonate hydroxyapatite (CHAMINO) microspheres to control drug delivery for the targeting of alveolar bone repair [201]. The results showed that the implantation of the microspheres showed significant new bone formation after 42 days, indicating that the composite microspheres have great potential in the clinical application of bone regeneration.

### 4.4 Arthritis

Arthritis is caused by inflammation, infection, trauma, degenerative lesions, and autoimmune factors such as joint inflammation. Common arthritis diseases include rheumatoid arthritis, infectious arthritis, osteoarthritis, and gout arthritis, etc. The main clinical manifestations are joint pain, swelling, deformity, and dysfunction, and some arthritis cases can lead to the loss of activity function, seriously affecting the patient's health and quality of life. The treatment of arthritis is often based on oral drugs [202], joint cavity injection, and physical rehabilitation [203], but these treatments can only relieve the pain and delay the course of the disease. They cannot achieve the purpose of prevention and cure of the disease. It was found that targeted drugs can inhibit proteoglycan expression in chondrocytes, thereby alleviating the disruption of cartilage [204]. Based on this, microspheres represent a new method of dosage that has been applied and developed in the past 30 years. Its advantage is the ability to prevent the drug from having various adverse effects in the internal environment and ensuring that the drug is released slowly, so that the drug can play a better role. At the same time, it also reduces the number and dosage of drugs, but also avoids the toxicity and side-effects caused by the systemic absorption of drugs. Therefore, the application of microsphere to treat arthritis is a promising area of study. For example, Laxmi et al. prepared vancomycin hydrochloride microspheres (VMSs) based on a Box–Behnken design (BBD) [205]. The optimized vancomycin hydrochloride microspheres (OVMSs) were 1.5 ​cm large. The in vivo antibacterial results showed that OVMSs could reduce infectious arthritis and bacterial load. Li et al. found that PLGA/chitosan/gelatin microballs loaded with hyperactivated platelet lysate (sPL) can provide effective and noninvasive repair of osteoarthritic articular cartilage [206].

### 4.5 Neural repair

Nerve injury includes central nerve injuries and peripheral nerve injuries [207]. Repair after nervous system injury has been a difficult problem, and drug treatment is one of the most commonly used methods to treat this injury. Neurotrophins act to promote neuronal survival and axonal regeneration, and can also promote regeneration in the central and peripheral nervous systems (Fig. 7C and D) [208]. However, in order to ensure the safe and efficient function of neurotrophic factors, treatment requires accurate and continuous delivery of drugs to the nervous system with the help of a drug delivery system. Microspheres have been used in these drug delivery systems to treat this type of damage because of their targeting ability and their ability to provide an efficicent and slow release. For example, Zeng et al. prepared nerve growth factor (TPP) ion cross-linked (NGF)-loaded chitosan/PLGA composite spheres (Fig. 7B) to study the release of NGF in peripheral nerve injury [88]. The results showed that the sustained release of NGF in vitro can not only promote the differentiation of PC12 ​cells but can also promote neurite growth. Moreover, TPP/chitosan/PLGA-NGF microspheres (Fig. 7E) can not only enhance sciatic nerve regeneration in rats, but can also prevent the atrophy of the rat gastrocnemius muscle. In short, these microspheres have great feasibility in relation to repairing nerve tissue.

### 4.6 Enteritidis

**Fig. 8:**
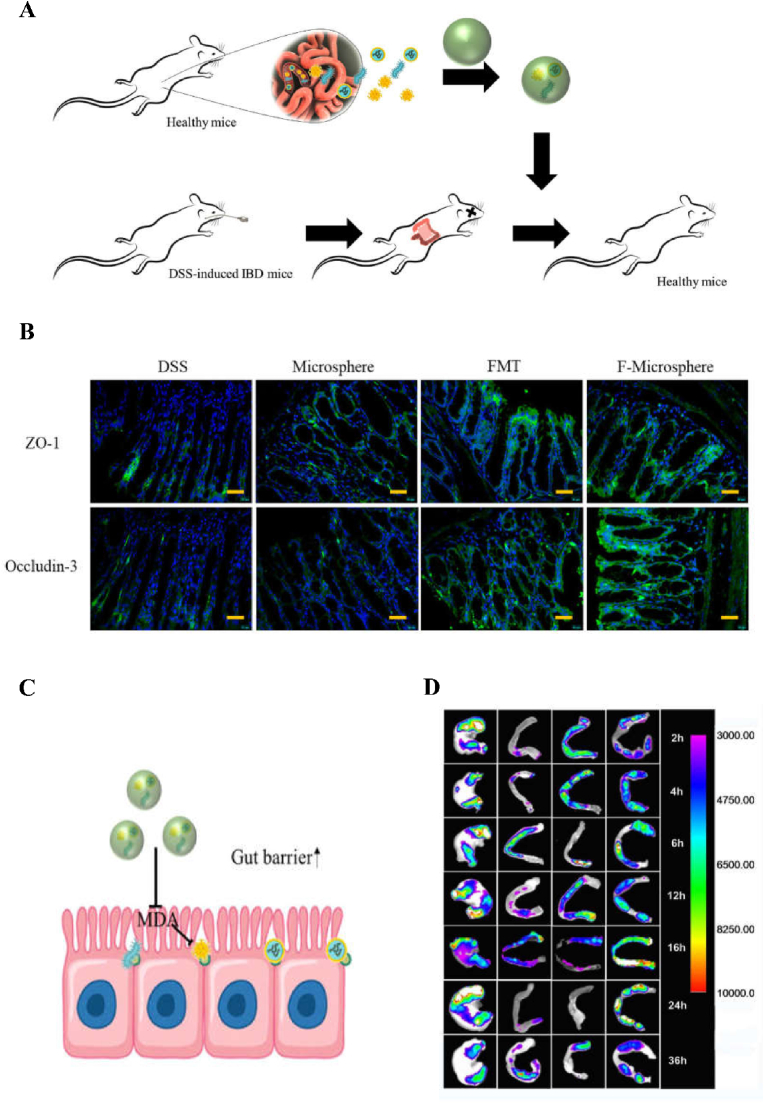
Therapeutic effect on protecting gut barrier. A Schematic of FMT in colitis rats. Faecal microbiota was fetched from healthy mice and transplanted to DSS-induced colitis mice. Reproduced with permission from Ref. [209]. Copyright 2020, Springer. B Fluorescent staining of ZO-1 and Occludin-3 in colon of mice in different groups. Scale bars are 50 ​μm. Reproduced with permission from Ref. [209]. Copyright 2020, Springer. C Schematic of the underlying mechanism of protecting gut barrier. Reproduced with permission from Ref. [209]. Copyright 2020, Springer. D The fluorescence intensity of FITC-CS microspheres in different residence time of different time (Left→Right:Stomach, Duodenum, Jejunum and Colon) varied from 2 to 36 ​h. Reproduced with permission from Ref. [210]. Copyright 2016, Elsevier.

Enteritidis refers to acute and chronic inflammatory lesions of the intestinal wall mucosa. The causes of enteritis are bacterial infection of the intestinal tract, poor diet, overeating, or the consumption of strong irritants or non-digestible food. According to the duration of the course of enteritis, enteritis can be divided into two types: acute enteritis and chronic enteritis. The former refers to inflammatory lesions of the acute intestinal wall mucosa, usually caused by bacteria, viruses, and other pathogenic microorganisms, and the latter refers absorption disorders and chronic intestinal wall mucosa inflammatory lesions, with an influence on the intestinal environment and with clinical manifestations of abdominal pain, diarrhea, and fever symptoms, seriously affecting the patient's quality of life and increasing their economic burden. Drugs are usually used for clinical treatments, but the effects of this treatment are not ideal because it can lead to adverse reactions and high drug resistance. In recent years, the use of microspheres as carriers of loaded drugs as a novel treatment for this disease has been reported. For example, Zhen et al. reported on the effects of fecal-coated alginate microspheres on sodium dextran sulfate (DDS) induction in relation to colitis in mice (Fig. 8A–C), demonstrating that fecal microbiota micropellets may be a potential treatment for enteritis and help to reduce intestinal barrier damage in colitis [209]. Wang et al. investigated the protective mechanism of icariin-loaded alginate-chitosan microspheres against colon mucosal damage in rats (Fig. 8D) [210]. In vitro release results showed that the number of icariin microspheres was higher in the simulated colon fluid than in the simulated gastric fluid and remained in the colon for more than 12 ​h, also indicating that these microspheres can achieve colonic protection by alleviating the inflammatory response.

### 4.7 Reproductive disease

People's living environments, habits, and work pressures are constantly changing with time, and people are increasingly facing reproductive problems. At present, medical technology is developing rapidly, and there are many means to treating reproductive problems, with drug treatment among the commonly used methods. Microspheres are often used as carriers for drug transport because of their good biocompatibility and sustained-release properties. For example, the clinical use of leuprolide microspheres for ovarian cysts before laparoscopic surgery is thought to be effective in improving the therapeutic effect [211]. Furthermore, microspheres can also be applied to treat reproductive problems such as infertility. For example, Mansouri et al. explored the ability of mouse embryonic stem cells to differentiate into putative primordial germ cells (PGCs) in alginate and alginate-collagen IV microspheres (CAMs) [212]. The results showed that the differentiation potential of ESCs to putative PGCs in CAMs was higher compared with the control group, and CAMs showed great potential in the differentiation of PGCs and in the treatment of infertile adults and, thus, may be a reliable means of producing mature germ cells.

## 5 Conclusions and prospects

In this study, we have introduced some of the research advances related to sustained-release drug microspheres in recent years. First, we introduced the role and types of drug delivery systems, discussing the concepts of sustained-release drug carriers and microspheres. According to the nature of the wrapped drug and the carrier material, microspheres with different microstructures can be prepared, with different scopes of use. We then summarized the methods used for preparing microspheres in order to meet different needs and applications, outlining the variety of methods applied to the preparation and processing of microspheres. Next, we summarized the materials used for the preparation of microspheres, according to the nature of the packaged drug and the application, explaining that the carrier materials can be natural or synthetic polymeric materials. Finally, we summarized the application of sustained-release drug microspheres in diseases, including tumor treatment, bone repair, nerve repair, etc.

In conclusion, sustained-release drug microspheres, as a kind of sustained-release drug carrier, have broad development prospects. However, there are some limiting factors in the development of sustained-release drug microspheres. Microspheres with different microstructures still have some deficiencies and need to be optimized. For example, the initial burst concentration of solid microspheres is high, the encapsulation rate is low, and the pore-forming agent added to the porous microspheres is difficult to remove. The methods used for preparing and processing microspheres have both advantages and disadvantages. For example, the hard template method requires the use of a large number of organic solvents, which are difficult to remove, can easily cause biological pollution. It is necessary to constantly explore ways of improving the size of the beads, their monodispersity, encapsulation rates, drug delivery ability, and industrial production, etc. In future research, continuous development in the areas of materials science, biomedical engineering, organic chemical industry, etc., can help us better solve the problems associated with the current sustained-release drug microspheres. We hope that the application of sustained-release drug microspheres in tissue engineering will be further expanded and the role of sustained-release drug microspheres will be broadened with the development of various disciplines.

## Credit author statement

**Qizhuang Lv**: conceptualized and planned the manuscript. **Lian Ruan**: wrote the original draft of the paper. **Mengrong Su**: wrote the original draft of the paper. **Xinyun Qin**: wrote, reviewed, and edited the original manuscript. **Qingting Ruan**: wrote, reviewed, and edited the original manuscript. **Wen Lang**: performed the literature search. **Minhui Wu**: performed the literature search. **Yujie Chen**: performed the literature search.

## Declaration of competing interest

The authors declare that they have no known competing financial interests or personal relationships that could have appeared to influence the work reported in this paper.
